# Exploring how Syrian women manage their health after migration to Germany: results of a qualitative study

**DOI:** 10.1186/s12905-021-01193-9

**Published:** 2021-02-02

**Authors:** Salma Kikhia, Ghaith Gharib, Alexandra Sauter, Natalia Caldeira Loss Vincens, Julika Loss

**Affiliations:** 1 Medical Sociology, Regensburg University, Regensburg, Germany; 2 Faculty of Medicine, Ulm University, Ulm, Germany; 3 Department of Public Health and Community Medicine, Gothenburg University, Gothenburg, Sweden; 4 Department of Epidemiology and Health Monitoring, Robert Koch Institute, Berlin, Germany

**Keywords:** Syrian women, Immigrant health, Germany, Access to healthcare, Post-migration stress, Resilience

## Abstract

**Background:**

In the recent years, the number of Syrians living in Germany increased drastically due to the massive displacement caused by the Syrian conflict. Syrian migrant women in Germany are challenged by both the migration process and the changing of social roles. Seeking out healthcare may be hampered by linguistic and cultural barriers, but the new context may offer opportunities for health and well-being (free access to health care, civil/human rights). Little is known about how Syrian women manage their health after their resettlement in Germany.

**Methods:**

In depth interviews in Arabic were conducted with 9 Syrian women who were recruited through purposive sampling (18–55 years, migrated in 2011–2017, different education levels), focusing on capabilities to control one’s health and to navigate the German healthcare system, and social/environmental barriers and facilitators to effectively manage their health. Interview transcripts were analysed using qualitative content analysis.

**Results:**

The women reported their health to be impaired by post-migration stressors, such as perceived discrimination, loss of social status and worrying about the future. Many interviewees felt disempowered and incompetent to successfully and actively navigate the German healthcare system, lacking information and not understanding their rights and options under the health insurance plan. The language barrier added to feeling vulnerable. Many women experienced doctors declining to treat them for capacity reasons; when treated, they often did not feel taken seriously or were dissatisfied with the emotional/cultural aspects of care. If possible, Arabic doctors were sought out. Some women, however, described improved resources for health, and appreciated better women’s rights as a source of power.

**Conclusions:**

The lack of information about the structure and offers of the German healthcare system, language and culture specific barriers as well as socio-cultural challenges are undermining the ability of Syrian women to manage their health effectively after their resettlement in Germany. Providing tailored information on the German healthcare system, creating a health-literacy supportive environment, and improving cultural sensitivity in healthcare provision could help Syrian women better utilize medical care offered in Germany.

## Background

Relocation to another country often poses serious psychological, Social, and familial challenges, demanding extensive individual adjustments, often resulting in health complications [1].

Given the recent massive displacement caused by the Syrian conflict (6.3 million in 2017), around 1 million individuals have reached Europe after crossing the Mediterranean Sea [2]. Germany, with its recent open border policy, received the largest number of Syrian migrants in Europe, increasing the number of Syrians living in Germany from 30,133 in 2011 to 746,000 in 2019 [3]. This makes Syrians the third-largest foreign population group in Germany [4]. Roughly half of this population group is female [5].

There is an information gap regarding the Syrian immigrant health situation and their specific needs, with even less gender-specific data available [6, 7]. Research shows that migrant populations from other countries tend to underutilize health services, engage in risky health behaviour and fail to receive timely healthcare. A variety of reasons account for these phenomena, including deficits in education, low income, lacking awareness of available resources and linguistic/cultural barriers [8]. Moreover, the specific situation of forced migration poses further challenges to the migrants’ health [9].

On a European level, the appropriate management of newly arrived migrants’ health problems is affected by barriers to access to health care including legal, communication, cultural and bureaucratic difficulties. This varies widely across Europe due to policy differences between health care systems and social services [10].

Despite a general lack of knowledge regarding the health status among Syrian immigrants in high-income countries in Europe in their post-resettlement phase, insights could be gained from a recent study on the health-related quality of life among Syrian refugees resettled in Sweden. Mental health problems constituted the most common health problems among this group, whereby women had an increased risk of experiencing physical and mental problems compared to men [11].

In Germany, a recent survey among refugees from Syria, Iraq and Afghanistan showed that this group had high prevalence of violence-exposure and traumatization prior to their arrival in Germany. They demonstrated high levels of depression signs and engaged in more unhealthy behaviours compared to the German population. Despite their various health complaints, they reported a lower use of medical offers, and low satisfaction and recovery after treatment. The language barrier was perceived as a big challenge, resulting in major difficulties in communicating with healthcare providers, looking for health services and understanding health information [12]. Other studies indicated that Syrian women are particularly vulnerable before and during the displacement with high probability of gender-based violence experiences [13]. A focus group with Syrian women in Germany identified a number of post-migration stressors, such as living conditions with limited privacy in shared accommodation centres, unmet needs for treatment of health complaints, and worries about their children’s wellbeing [14].

Women in this group, coming from a family-oriented culture, are often expected to be the caretaker of the family and to protect family values, culture and beliefs. This makes them more susceptible to post-migration stressors when they cannot meet these expectations [15]. Moreover, adapting to their new lives in Germany might add new challenges and expectations to their traditional roles. Still, one might speculate that the new context may offer Syrian migrated women opportunities for health and well-being, e.g. due to free access to healthcare and the condition of civil and human rights [16, 17]. Hence, it is uncertain how social and environmental circumstances act as barriers and/or facilitators to the ability of Syrian immigrant women to manage their own and their families’ health in Germany.

To the best of the authors’ knowledge, there are no studies that focus on how Syrian women experience managing their health and the health of their families after resettlement in Germany, particularly those living in decentralized accommodations, as those few studies cited above were performed among residents of refugee accommodation centres. Although it is expected that the women’s health situation would improve after moving out of the accommodation centres [14], this might make them harder to reach with health initiatives. Once they move to their private accommodation, they are also expected to navigate the German healthcare system on their own. This might pose new challenges.

Since mothers in the Syrian culture are the main caretaker of the health of their families and children, we believe that by looking at how women with a family (in this case, mothers) manage health situations, we can draw conclusions about the health situation of the entire family and in the greater sense the Syrian community in Germany. This may help inform the political, administrative and organizational efforts directed at the integration of this substantial population group.

The above described situation raises the research question guiding this study: “How do Syrian immigrant women experience managing their own and their families’ health, after their settlement in private accommodation in Germany?”

The objectives of this study are:

1. to describe how Syrian immigrant women in Germany feel about their capabilities to manage their health and that of their families.
2. to explore the social and environmental circumstances of Syrian migrated women, that act as barriers and/or facilitators to effectively manage health.

## Methods

### Study design

This research used a qualitative design in which in-depth interviews with Syrian immigrant women were conducted. A qualitative approach was chosen as the appropriate method since it allows to understand how the participants construct their own meaning of reality, which corresponds with the exploratory nature of this study [18, 19]. Interviews allow an in-depth understanding of the participants’ lived experiences and perceptions and offer the possibility to address sensitive matters in a confidential setting [20].

### Study setting

The study was conducted in the federal republic of Germany, a high-income country with 83 million inhabitants [21]. The German healthcare system is based on compulsory statutory health insurance and is highly decentralized [22].

Most Syrians who entered Germany after 2011 were asylum-seekers, due to the huge migration wave after the beginning of the Syrian conflict [5]. Once they seek asylum in Germany, people are obliged to stay in “initial reception-centres” for up to 6 months [23]. These centres provide an initial health assessment and very basic health services [24]. Afterwards, asylum applicants are distributed across the 16 states of Germany based on a distribution quota system and are moved into collective accommodation centres. During this stage, access to healthcare is restricted to emergency care and acute medical cases for the first 15 months of residency in most parts of Germany and requires the issuing of a health-voucher from the social-welfare office [25, 26]. Once the decision of granting protection-status is made, refugees gain access to the same healthcare benefits as German citizens and are not obliged to stay in accommodation centres [24, 27]. However, refugees often face difficulties finding private accommodation [28].

Syrian refugees start the integration process by visiting government-run “integration courses”. These courses provide the newly arrived immigrants with basic knowledge of the German language and information about life in Germany, covering aspects of social life, employment-seeking and cultural norms, without a specific focus on health [29].

Initially, the capital city of Berlin was chosen as the study setting, since it was thought that the capital’s large proportion of migrant populations, including Syrians, would facilitate the sampling. However, it was later suspected that the experience of Syrian women living in Berlin might differ from those living elsewhere in Germany, possibly due to higher access to facilities or networks with the highest centrality of the capital, which might not be common to other German cities or rural areas. In an attempt to capture the diversity of the Syrian women’s experiences in Germany due to the location, this was later expanded to the cities of Stuttgart, the capital of the western German state of Baden-Württemberg and Leipzig, the most populous city in the eastern German state of Saxony. Additionally, efforts were made to account for experiences in rural settings; and therefore, Zossen, a small town in the state of Brandenburg was also included in the sample.

### Sampling and recruitment of interviewees

The study population were Syrian women, who migrated to Germany within the last immigration wave. The term “immigrant” refers here to Syrian women who moved to Germany after the beginning of the war in Syria in 2011, regardless of whether they were seeking refuge, reuniting with displaced family, or moving for other purposes.

The eligibility criteria for participation in this study were to be a Syrian woman, a mother, aged between 18 and 55, migrated to Germany between 03.2011 and 12.2017 and living in a private accommodation.

The recruitment was done via purposeful sampling to allow for maximum variation in experiences [20]. The purpose was to reach women in different age-groups and with different socio-economic, marital and educational backgrounds. Attempts were also made to interview both veiled and unveiled women, considering that veiled women may have different experiences in Germany than unveiled women. The interviewees’ characteristics are detailed in Table 1.

**Table 1:** Study sample

School Education	Number	Veiled	Mean age	Language level	Mean number of children	Marital status		City
In years				A,B*		Single	Married	Berlin-Leipzig Stuttgart-Zossen**
7–9	n = 2	yes = 2, no = 0	33	A = 2, B = 0	2	0	2	Berlin = 1 Zossen = 1
10–12	n = 2	yes = 0, no = 2	42	A = 0, B = 2	4	1	1	Berlin = 2
> 12	n = 5	yes = 3, no = 2	42	A = 1, B = 4	1.4	1	4	Berlin = 3 Stuttgart = 1 Leipzig = 1
total	n = 9	yes = 5, no = 4	40	A = 5, B = 3	2.1	2	7	Berlin = 6 Other = 3

*Language level A is beginner, B is advanced. None of the interviewees had level C (fluent)

**Berlin population 3.613 million [30], Leipzig population 596,517 [31], Stuttgart population 623,738 [32], Zossen population 17.957 [33]

The interviewees were recruited through various channels; organisations, social media networks and the first author’s personal contacts with Syrian individuals and professionals (i.e. doctors) who acted as gatekeepers. The first author (SK) approached women personally through these different channels to raise their interest in participating in this study. From originally 13 women interested in participating, 9 women could be interviewed due to the limited timeframe for data-collection. All women included in this study had access to the full scope of services of the state health insurance*,* as they had lived more than 15 months in Germany.

### Data collection and informed consent

In total, nine individual in-depth interviews were conducted in January–February 2019 by SK. The study’s strict time schedule was due to it being part of a master’s thesis requirement.

After being initially approached, the study was explained to the women in written and oral form, and if they agreed to participate, a suitable date and place for an interview were set. Two of the nine interviews were conducted through video-call and the rest in person. The interviews were conducted in a place that was deemed favourable for the interviewee. Sometimes the first author visited the women in their houses, other times the interviews took place in a public place like a café or a park. Written consent to be interviewed and audio-recorded was obtained in all cases. All participants agreed that excerpts of the interviews will be cited in scientific publications with all information which could lead to an identification of the interviewed person (IP) being changed or removed to ensure that the resulting overall context of events cannot lead to an identification of the person.

A semi-standardised interview-guide was developed specifically for this study and used to conduct the interviews. The interview guide was tested with two pilot interviews that were later included in the analysis. The focus of the interview guide was on capabilities to control one’s health, resources, access and information about the German healthcare system and social/environmental barriers and facilitators to a healthy life. The interview guide developed for this study is provided as Additional File 1. The interviews took 60–90 min and were performed in Arabic, the mother tongue of both the interviewing researcher and the interviewees.

### Data analysis

The interviews were transcribed verbatim and translated into English. The transcripts were analysed using qualitative content analysis as described by Graneheim and Lundman [18]. The analysis followed an inductive approach, whereby issues that the women considered of greatest importance to them were lifted to create the subcategories and categories. First, the transcripts were read and re-read several times to achieve an understanding of the content of the interviews. The transcripts were then coded by the first author (SK), assisted by the software Atlas ti 7. The codes were then discussed with GG and upon reaching consensus, a codebook was created. SK then grouped the codes into subcategories that were condensed into categories upon discussion with the other co-authors. A higher level of abstraction was achieved by the construction of sub-themes and themes. This was accomplished through a process of feedback and discussion with the co-authors (JL, NV) who continuously reviewed the categories and emerging themes. Differences in the categorization of the researchers were discussed within the research team until consensus was reached.

### Ethical aspects of the study

The study was approved by the Ethics Committee of the University of Regensburg (19-1319-101). The participants were fully informed both in verbal and written form about the study procedure and written consent was obtained from the interviewees prior to the interviews. Interviewed women received no incentive and participated voluntarily.

## Results

Five main themes emerged in the interviews: (1) “post-migration stressors impacting health negatively” (2) “Feeling dissatisfied with the medical, cultural and organisational aspects of healthcare”, (3) “Being vulnerable because of the language deficiency”, (4) “Lacking the information, experience and agency to navigate the healthcare system”, (5) and (6) “having chances for resilience”. Table 2 demonstrates an overview of themes and sub-themes.

**Table 2:** Overview of themes and sub-themes

Theme	Sub-theme	Anchor quote
Post-migration stressors impacting health negatively	Lack of social support	“I’m completely on my own.…I take care of [my son], but it’s a big problem when I get sick. There’s no one to take care of me” (IP9)
	Experiencing discrimination	“I feel like people avoid … helping me since I wear hijab. That’s why I don’t feel comfortable going with my son to see the doctor, and I let his father take him” (IP6)
	Loss of social and professional status	“They won’t recognise my university degree from Syria, so I probably won’t have a chance of finding a good job (..) This is a huge source of worry in my life” (IP9)
	Maintaining and changing social roles	“I neglect doing things for my wellbeing and prioritise things for the wellbeing of my family.” (IP2)
Feeling dissatisfied with the medical, cultural, and organisational aspects of healthcare	Perceived problems in accessing healthcare	“He didn’t see her [my daughter], because he had no capacity for new patients (..)Until today we can’t find a doctor for my daughter.” (IP4)
	Perceived effectiveness and quality of medical care	“In Syria, they would’ve at least taken care of her [my daughter’s] pain. But here, they just sent her home and told her to see a specialist” (IP5)
	Medical mistrust	“I wish I could find this one doctor that I can trust and be fully confident in what he does and says. But when I don’t see results, when my and my family’s health problems and symptoms don’t get better, I only get frustrated and sceptical about healthcare here “(IP6)
	Cultural barriers in healthcare communication	I didn’t understand the coldness with which she (her gynaecologist) treated me, and she clearly didn’t understand my nature either, we had no channels for proper communication. (IP8)
Being vulnerable because of the language deficiency	Feeling powerless due to the language barrier	“It’s like having a handicap” (IP3)
	The language barrier limiting access to healthcare	“ I was advised to see German doctors, but I can’t because I don’t know the language (…) I was told that this doctor is the best option available to me in Arabic, so I’m going to continue treatment with him even though I don’t see any improvement.” (IP4)
Lacking the information, experience, and agency to navigate the healthcare system	Lacking information about the healthcare system and offers	“The problem is that everything is in German (language), even when the target group is immigrants (…) I’ve been living here for two years and didn’t know that this family-centre in my neighbourhood exists. They could’ve helped me with my pregnancy or told me about the midwife thing.” (IP8)
	Lacking the necessary experience to navigate the healthcare system	I really don’t understand how the system here works. When I see a doctor, I just get transferred to another doctor, and I don’t feel like my case has been treated. I’m frustrated and have no control at all, the system is just impossible. (IP6)
	Reacting passively to unsatisfying healthcare	“I know that I have the right to speak up, because they [the health professionals] are getting paid for it, so I must receive the healthcare that I need. But (…) I don’t want to cause any troubles” (IP4)
Having chances for resilience	Experiencing liberating conditions for women	“In Syria, the entire system and society would’ve stood against me [and my decision to abort my pregnancy], but here, the law protects me.” (IP7)
	Having resources for better healthcare	“It’s an advantage that I can always afford taking my child to the doctor, and that the insurance covers everything. In Syria, as a single mother, my salary might not be enough to take my child to the doctor every time he gets sick.” (IP9)
	Receiving help/advice from locals	“I thought that there was nothing to be done about what happened [dental malpractice with high out-of-pocket payment] and that I had to bear with the unsatisfying results and costs. But thank God it was solvable, only because of the help of this woman [the social volunteer]” (IP2)

### Post-migration stressors impacting health negatively

The women described living in continuous stress in Germany, which they considered as the main factor that undermined the possibility of a healthy life.

I think it’s really difficult leading a healthy life with all the stress we’re exposed to daily. I mean I feel like I’m always nervous or angry at my kids for no real reason, because I’m not capable of doing the things I have to do with the limited time I have. (IP6)

Different reasons were given for the daily distress. Almost all interviewees described that they were overwhelmed with the German bureaucracy, which deteriorated their mental health. Some older women described themselves as lacking the skills to adapt to and navigate the German administrative system and civil services and attributed this to the later age of migration.

#### Lack of social support

Another determinant of chronic stress described by the women is the lack of social support, which impacted their health both directly and indirectly. Stripped of the help of their friends and families, the women explained that their responsibilities were now much bigger than they had been in Syria, and much harder to comply with, since the women must rely completely on themselves. This was especially difficult for single mothers.

We’re alone here, we lost all the social life that we had in Syria, that’s why it’s really hard for us here. I feel alienated and I don’t have anyone to talk to or ask for help (IP4)

I’m completely on my own. …I take care of [my son], but it’s a big problem when I get sick. There’s no one to take care of me [then], like that time when I had a back-pain crisis for 5 days and couldn’t move. My son was still a baby and needed diapers and milk, and I had no one to ask for help (IP9)

#### Experiencing discrimination

The experience of hostility and discrimination because of being a refugee or foreigner interfered with women’s everyday lives and wellbeing, especially for those wearing hijab.

I feel like people avoid … helping me since I wear hijab. That’s why I don’t feel comfortable going with my son to see the doctor, and I let his father take him (….) I can’t go to a gym because I’m scared of going out in the evening, especially after the incidents with verbal and physical violence that I experienced. (IP6)

#### Loss of social and professional status

For women from Syrian upper middle-class, or with a higher academic degree, their loss of social status in Germany and perceived social stigma of being an immigrant, or a refugee, was described as “a tremendous stressor” and “disempowering” by itself. (IP8)

They won’t recognise my university degree from Syria, so I probably won’t have a chance of finding a good job. What job can I find? A cleaning lady? This is a huge source of worry in my life. Sometimes I wake up at 2 am with a striking headache that would last for days. I know it’s caused by stress. (IP9)

Some women also described mental ill-health as a result of worrying about an uncertain future, since they didn’t know whether they would be allowed to stay in Germany.

#### Maintaining and changing social roles

The women reported how trying to combine different roles in their lives in Germany wore them down physically and mentally. Adopting new roles, like full-time studying the language, and taking care of administrative paperwork, in addition to their traditional roles i.e. being fully responsible for childcare and household-chores, were said to exacerbate stress and leave them with no time to relax.

Some women admitted neglecting their own health, i.e. they did not allow themselves time to relax, or postponed seeking help for a chronic health problem. Being a mother meant prioritising their family’s wellbeing over their own. While the interviewees were aware of possible negative consequences for their own health, they generally endorsed this conduct as being normal and compliant with the role of a mother.

I always forget to drink water or look after my own health. I neglect doing things for my wellbeing and prioritise things for the wellbeing of my family. (IP2)

Interestingly, not much difference was seen in the narratives of women from different socioeconomic and educational backgrounds when it comes to post-migration stress they reported to experience. Veiled women were most susceptible to stress related to discrimination, while women with the lowest language acquisition were the most socially isolated. Availability of social support (from family members) seemed to be protective against exacerbating stress.

### Feeling dissatisfied with the medical, cultural and organisational aspects of healthcare

#### Perceived problems in accessing healthcare

The women reported problems in accessing (timely) healthcare, as the doctor’s offices’ capacities to receive new patients were claimed to be limited. Searching for a health professional willing and able to treat them or their children was very troubling for many interviewees. According to the women, appointments could only be scheduled very far in the future, often after a period of 3–6 months, during which they expressed having to put up with their symptoms and the uncertainties about their health situation or that of their families.

When we got here, my daughter got sick and we went to see a German doctor. He didn’t see her because he had no capacity for new patients. He gave us a transfer to another doctor. My husband went the next day, but they too were full (…) Until today we can’t find a doctor for my daughter. (IP4)

Sometimes, the inability to find an available healthcare provider prevented women from receiving healthcare. The difficulty in receiving timely appointments also sometimes resulted in fatalism.

My general practitioner gave me three transfer-notes, one to an endocrinologist, another to a cardiologist, because I had palpitations, and a third for my leg varices. I started calling and trying to make appointments with the different doctors, I was offered appointments in 8 and 9 months. I don’t find it logical to wait that long for an ECG-test. So, I ended up cancelling all three appointments. I don’t know what might happen in 8 months’ time, I could be dead by that time. (IP6)

#### Perceived effectiveness and quality of medical care

Most of the interviewed women criticised the medical treatment they received from doctors in Germany. They felt that their health complaints were not being taken seriously or that they were not properly examined or treated by the healthcare provider. This was often considered a sign of neglect, prejudice, or discrimination.

I have back pain, because I have had a desk-herniation since the time I was in Syria. I saw the doctor here when the pain exacerbated. He said: “It’s psychosomatic.” I told him that I have a diagnosis from Syria and that I've previously done radiographs that prove it, but he refused to believe me and said: “You Syrians have gone through a lot of stress”, so he didn’t even order an X-ray (IP8)

Like the time when my daughter was hospitalized (…) In Syria, they would’ve at least taken care of her pain. But here, they just sent her home and told her to see a specialist. She was in huge pain when I took her home. I don’t know if it’s discrimination against refugees. I don’t believe Germans would be fine with being treated like that. (IP5)

#### Medical mistrust

Some interviewees did not feel well informed by their doctors. This was sometimes portrayed as the reason for medication errors (mix-ups with the doses or types of medications). As a result, many interviewees had developed mistrust towards the medical care they received in Germany.

I wish I could find this one doctor that I can trust and be fully confident in what he does and says. But when I don’t see results, when my and my family’s health problems and symptoms don’t get better, I only get frustrated and sceptical about healthcare here (IP6)

#### Cultural barriers in healthcare communication

Many women also perceived a lack of empathy from the healthcare providers and criticized the emotional and cultural aspects of the healthcare they had received.

In Syria, you form a personal relationship with your doctor. Here it’s fast and systematic. This is related to the culture here…I didn’t understand the coldness with which she (her gynaecologist) treated me, and she clearly didn’t understand my nature either, we had no channels for proper communication. (IP8)

Consequently, many women resorted to trusted doctors back in Syria, often sent them medical tests and asked for their second opinions on an already-made diagnosis by their healthcare provider in Germany. In cases of disagreement, the women would often go by what their Syrian doctor would tell them.

### Being vulnerable because of the language deficiency

The interviewed women thought of the language deficiency as a big barrier to optimal healthcare in Germany, although the extent to which this barrier prevented them from receiving healthcare varied depending on their language skills.

#### Feeling powerless due to the language barrier

Not knowing the German language resulted in feelings of powerlessness and vulnerability when seeking out healthcare (“*It’s like having a handicap”, IP3).* The disrespect that some interviewees reported to have experienced was sometimes also attributed to the language barrier.

My daughter is still relatively new here and can’t speak German. I think that’s a reason why she’s being treated in an unfriendly and impatient way. She tries asking the doctors or nurses to speak English, but they just say they can’t. (IP5)

None of the women reported using the help of a professional translator in healthcare settings. Reasons given by some women for this were perceived unavailability of such services or these being inaccessible to the women in their living areas after they moved to living in the community. Instead, some women resorted to relatives or friends with better German language abilities. This leads to the feelings of dependency and guilt.

In Syria, I could make a simple phone call and get an appointment the next day. Here, I’m still responsible for me and my family, …but I feel powerless and dependent on others to meet these responsibilities. I have to rely on my son because he can speak the best German in the family. And therefore, he struggled academically, because he had to commit a lot of time to helping us” (IP6)

#### The language barrier limiting access to healthcare

Women who neither speak German nor English reported that they are restricted to seeing Arabic-speaking doctors. Due to the shortage of Arabic-speaking doctors, these women need to travel longer distances, wait longer for an appointment, and have less alternatives if not satisfied with the received care.

I don’t have any other options. I was advised to see German doctors, but I can’t because I don’t know the language, and there are no translators to help. I was told that this doctor is the best option available to me in Arabic, so I’m going to continue treatment with him even though I don’t see any improvement. (IP4)

### Lacking the information, experience and agency to navigate the healthcare system

#### Lacking information about the healthcare system and offers

Most of the interviewed women explained that they were not adequately informed about existing health services (e.g. midwife services during pregnancy), so they missed out on utilizing these offers. Although all interviewed women expressed the desire to participate in preventive health activities, most women were convinced that there are no such available offers in their neighbourhoods, or only found out about them very late. Some pointed out that health offers are not properly communicated to immigrant families.

The problem is that everything is in German (language), even when the target group is immigrants. If an organisation has brochures in Arabic, but only inside the actual organisation building, how would people who don’t know about it, find out that it offers services for them? I’ve been living here for two years and didn’t know that this family-centre in my neighbourhood exists. They could’ve helped me with my pregnancy or told me about the midwife thing. (IP8)

In addition to lacking information about existing services, some women seemed not to know about their health rights and options under the health insurance plan. This sometimes resulted in confusion and unnecessary out-of-pocket payment.

#### Lacking the necessary experience to navigate the healthcare system

The women frequently reported feeling lost inside the healthcare system, which was described as “too complicated” and “inefficient”. The lack of transparency results in feeling helpless, at the mercy of the system, and frustrated.

I really don’t understand how the system here works. When I see a doctor, I just get transferred to another doctor, and I don’t feel like my case has been treated. I’m frustrated and have no control at all, the system is just impossible. (IP6)

Unlike when they were in Syria, the women noted having limited access to other people’s experiences due to a more limited social network, mostly consisting of other inexperienced immigrants. Many women relied on social media for recommendations and orientation, which often provided inaccurate information.

There is no real choice in this. I have no experience that allows me to make an informed decision, because I don’t know which doctor is good, and I have no one to ask… When I’m in my own country, I know how to inform myself. …but here I have no idea. (IP8)

#### Reacting passively to unsatisfying healthcare

Although many women were frustrated and irritated about inefficient, non-empathic healthcare, or lack of transparency, almost none took any (legal) action to complain about limited access, malpractice, or failures of the system. Different reasons were given by the women to explain why they put up with the perceived grievances in a passive way (see Table 3).

**Table 3:** Reasons given by women for reacting passively to unsatisfying healthcare

Sub-category	Sample quote
Lacking the energy and expertise	“I know the importance of complaint mechanisms when things are not working well (…) but turning to the law takes a lot of time and energy that I don’t have. I don’t know the legal system here; one needs to be very acquainted with the way things work in the country to run this procedure… When you’re an immigrant, you’d rather give up and live with the situation” (IP8)
Powerlessness and lack of courage	“I know that I have the right to speak up, because they [the health professionals] are getting paid for it, so I must receive the healthcare that I need. But I’m shy and don’t know how to speak up for myself. I don’t want to cause any troubles” (IP4)
Distrusting the utility of complaints	“No, I haven’t thought about filing a complaint. How would that benefit me? Just complaining for the sake of complaining, this won’t change anything” (IP6)

### Having chances for resilience

#### Experiencing liberating conditions for women

Enjoying better rights as a woman in Germany was mentioned as a source of power by some women. These women felt that the laws in Germany are in their favour and encouraged them to act as independent individuals, which according to one interviewee “brings peace and power from within” (IP1). This was especially evident in the case of single mothers, who described having sovereignty and a sense of empowerment because of favouring laws (e.g. custody for their children, financial support) and an improved social position of women.

Our society was very harsh on women, now I’m far away (…) The most important thing is that I’m financially independent here. This is what makes a woman strong, because she doesn’t have to rely on any other man. (IP5)

The most important thing in the world for me is that I have custody for my child (…) even if I’m divorced or windowed, no one can take the children away from the woman, even if she remarries. This is so important, and I cherish it so much (IP9)

As a result, some women were able to negotiate their health needs or wishes in face of traditional or religious norms. For example, one woman was able to pull through the decision to abort an unwanted pregnancy, against the traditional views of her husband and family.

In Syria, the entire system and society would’ve stood against me [and my decision to abort my pregnancy], but here, the law protects me. My family doesn’t know about this, because they’ll criticize my decision, just because of social norms and traditions. (IP7)

#### b. Having resources for better healthcare

The women appreciated that the health insurances covered their health care costs and thought of it as allowing them and their families better chances of healthcare.

It’s an advantage that I can always afford taking my child to the doctor, and that the insurance covers everything. In Syria, as a single mother, my salary might not be enough to take my child to the doctor every time he gets sick. (IP9)

In addition, the women who were able to benefit from the services for pregnant women, praised them for being “very supportive and optimal”, especially in situations when they had no other source of social support.

One good thing about Germany is that the healthcare provided for pregnant women is very well-cared for. I was thoroughly monitored by my gynaecologist and very closely observed in the hospital. When I was discharged, I was assigned psychological support since I had preterm delivery, in addition to physiotherapy, and a social worker who helped me with the household (…) Back then my husband was away, and I was entirely on my own, so if it wasn’t for these services, it would’ve been really difficult. (IP1)

#### Receiving help/advice from locals

Few women narrated situations in which German people, be it professionals, social volunteers or acquaintances helped them orient themselves inside the healthcare system, overcome obstacles or even reclaim their right in healthcare. For example, one interviewee was able to retrieve treatment costs after an incidence of dental malpractice through the dedicated help of a German social volunteer.

I knew her [the social volunteer] from the social services and when I told her she told her husband who knew his way around these things, maybe he is a lawyer (…) I thought that there was nothing to be done about what happened [dental malpractice with high out-of-pocket payment] and that I had to bear with the unsatisfying results and costs. But thank God it was solvable, only because of the help of this woman. (IP2)

## Discussion

### Main findings

The Syrian immigrant women in this study reported that they experienced a wide range of obstacles in managing health after resettlement in Germany, like difficulties accessing healthcare and in communicating effectively with healthcare providers. Some felt incapable of navigating the complex healthcare system, because they lacked information on the existing services, and their rights and options in healthcare in Germany were often not transparent to them. The interviewees were frequently unsatisfied with the healthcare experiences they made—for medical and cultural reasons. Since some women continuously found themselves in situations where their efforts of seeking healthcare would not result in satisfying results, they felt a lack of control and many eventually gave up on trying. Many women developed mistrust and passivity towards health system-related situations. The language barrier added to feeling vulnerable. All women perceived that their health was impaired by various post-migration stressors which undermined the possibility of leading a healthy life in Germany. They also struggled both with maintaining their traditional social roles and with combining those with new roles that the new setting imposes. These barriers to health were shared by women across different socioeconomic and educational status and varying language levels. On the other hand, some interviewed women described that their health resources had improved in Germany, e.g. public health insurance and appreciated better women’s rights as a source of power.

### Comparison with other studies

One major finding of this study is that Syrian immigrant women lacked information about the structure of the German healthcare system and experienced difficulties with the access to healthcare. This is in line with results from a mixed-method study among German health professionals [34]. Those experts reported that many (Syrian) refugees were not familiar with many structural aspects of healthcare, such as the separation between outpatient and inpatient systems, the role of general practitioners as gatekeepers to specialist care, the health insurance costs-coverage schemes and the principle of appointments and waiting-times.

This is also in line with results from a qualitative study and literature reviews on the barriers related to access to health services for refugees in Europe and OCDE countries (The Organisation for Economic Co-operation and Development). Results show that refugees’ and migrants’ lack of knowledge of entitlements and available services are the major obstacles to accessing healthcare and using services appropriately. Specifically, it is demonstrated that refugees and migrants showed a poor understanding of primary healthcare and the appointment system. Like our findings, identified problems were the absence of health service information for migrants upon arrival in the country and difficulties in navigating the health system [35].

What could be regarded as new findings of our study is the extension of these findings to grasp the lived experience of the users i.e. the Syrian immigrant women in Germany. The interviewees in our study could not disentangle what exactly they were doing wrong in the healthcare system, nor what exactly they did not know or understand. Their constant failures in reaching satisfying healthcare was interpreted as inefficiency of the healthcare system itself. On the other hand, many interviewed women internalized these failures, resulting in a what has been described as “learned helplessness” in dealing with the healthcare system and providers [36].

A qualitative evaluation study of native-speaking health counselling services designated for Turkish and Russian immigrants in Germany illustrates that effective healthcare services usage by immigrants is hampered by a series of barriers that are similar to those found in our study. These include language problems that result in low literacy and limited ability to express oneself and ask questions in health settings. In addition, these groups of immigrants were found to have information deficits about the German healthcare system, its offers, and the modalities of its use, which limited their access to health and counselling services [37]. Our findings are also comparable to those from other European countries (for example, Italy, Sweden and Cyprus) that show that communication, information and health literacy issues result in poor health service provision for migrants, as one scoping review of the European literature demonstrates [38].

The women in our study often described being unsatisfied with the cultural aspect of care and found it difficult to establish trust with their healthcare providers. They often perceived the treatment from doctors to be insensitive, and sensed stereotyping and prejudice in healthcare settings. Similar findings have been reported by other immigrant groups in Germany, e.g. Pakistani women, whose birth experiences in Germany were greatly affected by health professionals’ attitudes which was perceived to be culturally insensitive [39]. Several European studies have highlighted cultural-specific barriers in the health care utilisation and access among refugees and immigrants [40, 41]. In a 2016 qualitative, comparative study in seven European countries on health needs, barriers and wishes of refugees, there was a clear wish expressed on part of the refugees for cultural competence in healthcare provision [42].

This study also highlights the socio-cultural challenges faced by Syrian women during their resettlement process in Germany, and how these compromise their mental health. Many of the described “post-migration stressors” confirm findings in the literature on refugee and immigrant women's’ health during the post-migration phase in general [15], and in the German context [43], although those studies have not been performed among Syrian women.

### Implications for policy, practice and research

Our findings showed that the women lacked the ability to understand and navigate the complex healthcare system, which is part of health literacy [44]. There is an abundance of international literature indicating that people with migration backgrounds are often limited in their health literacy, compared to the native-born populations, resulting in inequalities in health services-use and health outcomes [45, 46].

In the light of the seemingly existing confusion and helplessness in the healthcare-seeking behaviour of Syrian immigrant women, it seems critical to broadly address this group with services that make them more familiar with the structure of the German healthcare system and its available services. Although several health-related initiatives have been developed to provide different immigrant groups with culturally-sensitive health counselling (REFUGIUM,^1^ MiMi,^2^ UPD^3^), many of these initiatives are time-limited and are not part of the standard care, which might keep immigrant segregated in the type of care they receive in Germany. Additionally, the migrants’ unawareness of these services remains the main barrier to their use [37]. Therefore, it is paramount to communicate these health services more effectively to Syrian immigrants, e.g. by using the Arabic language and advertising them through channels relevant to the target group (ex. Arabic newspapers and networks in Germany). It could also be helpful to include health system orientation materials into the curriculum of integration courses for newly-arrived immigrants. On the system level, effective networking in and outside health care services is critical for integrated care for immigrants. This includes supporting interdisciplinarity, networking and coordination between primary care services, social services, and migrant-specific health care services.

The women in this study perceived reluctance from healthcare personnel to provide enough explanation of their health situations. Although they were looking forward to taking an active role in health-related decisions and improving their health information skills, they perceived that there were lingual and/or cultural considerations on the health professionals’ side that affected the quality of patient informing practices. The concept of a supportive ‘health literacy environment’ acknowledges that patients’ actions and understandings are influenced by the clinical setting and the attitudes and actions of everyone involved in patient care. Care environments, i.e. the general practitioner’s office, could be transformed to health literacy responsive organizations, e.g. by fostering communication and inviting questions by providing forms in an easy-to-read format and providing assistance with completing forms [49–51]. For patients with migration background, this approach also includes creating and improving cultural sensitivity and competence in healthcare settings. The reports of the interviewed women imply that many doctors and nurses were perceived to be impatient and not empathetic, which was put down to prejudice against migrants. This raises the issue of cultural sensitivity in healthcare provision for immigrant groups and warrants the promotion of the German concept of “intercultural opening” in healthcare provision, which implies reduction of communication barriers by using interpreters, understanding and mediating cultural differences, avoiding cultural stereotyping and promoting an open-minded, reflective professional approach [52]. For example, intercultural openness issues should be included in the curricula of medical programs for students as well as in continuous medical education of health practitioners in Germany. Moreover, efforts should be taken to fight discrimination, prejudice, and xenophobia both in the society and in healthcare provision. This should be addressed on a societal level to allow for true integration in the community, as well as on a systemic level through the implementation of policies against acts of discrimination in health care facilities.

In addition to addressing to challenges of healthcare access and cultural sensitivity, there seems to be a need to empower Syrian migrant women to overcome the passivity and helplessness in their health seeking behaviour in Germany. Empowerment is a key concept in (migrant) health promotion and should address not only aspect of healthcare provision (for example, provision of information in migrants’ own language about their rights and the functioning of the health and social care system) but also those of health determinants (for example, facilitating the opportunities to learn the language of the host country for migrant women [53]. Moreover, targeted outreach activities to build the capacity of Syrian immigrant women to effectively respond to challenges might be warranted. This is most viable on the intersectional level involving forging working relationships between health and diaspora organizations as well as women networks and empowerment groups.

The latter is particularly important, when considering, based on the findings of this study, that Syrian immigrant mothers in Germany are an extremely vulnerable group to mental distress in their resettlement process. The interviewees reportedly underwent a substantial loss of their resources in the migration (i.e. social and professional status, social capital). Moreover, basic psychological needs were reported to be greatly unmet in their living environment in Germany, like having a sense of belonging (alienation, lack of social support), feeling valued or appreciated by the society (stigma, discrimination) or having a sense of control over their destiny (uncertainties about the future). Our study also highlighted the issue of gender and social roles of this population group after migration, how these roles are negotiated throughout the resettlement process and how that impacts the health and well-being of the women. For example, the migrated women explained how they face time and energy strains while they try to adopt new social roles that correspond to the aspiration of the new society (financial and social sovereignty) while fully maintaining their traditional roles and family obligations. These findings alert to the need for addressing some problematic aspects of the integration of Syrian immigrant women in the German society on a policy level (for example, facilitation of recognition of former professional education, fighting discrimination), in addition to responding to their mental health needs through targeted mental health services for migrant women, and supporting migrant women to develop their social networks [54, 55].

The importance of the later points becomes more evident when looking at the resilience chances expressed by the women in our study. Resilience facilitators were identified in this study as important factors affecting the resettlement process and health of the Syrian immigrant women. Receiving social and professional support and advice from German professionals or friends helped women make oriented decisions in the healthcare system and navigate stressful conditions. In addition, non-health laws (like mother’s right to custody of her children) appear to have major implication on women’s health and empowerment. This highlights the importance of an intersectional approach that involves legal, social and organisational efforts in improving the health situation of Syrian immigrant women in Germany.

Hence, resilience chances should be given more weight in the integration and health discourse and interventions targeted at Syrian immigrant women, in an attempt to buffer the stressful demands. Resilience is a very dynamic and multidimensional process that enhances the individual’s ability to withstand, adapt to, and recover from stress and adversity [56, 57]. The women in this study perceived possibilities for health and well-being in their environment despite the stressful overall context: such as improvement in women’s rights, government resources like public health insurance and women health offers, and help from locals. However, these factors were seldom dominant in the women’s perception of their overall lives’ situation. Conversely, the positive influence of these factors was by no means equally shared by all the women, despite most of these factors being enforced on a policy level. Therefore, research is needed to explore how the resilience chances for this group could be better incorporated into health and integration efforts in a culturally-sensitive and empowering manner.

### Methodological considerations

This study was the first to be conducted in the German context to capture the experience of Syrian immigrant women with managing health after their resettlement in the society in Germany. Although it cannot be assumed that our findings are representative for the experiences of all Syrian women in Germany, the aim of qualitative studies is not to provide representative data, but to gain deeper understanding of social and psychological processes. The use of only one native interviewer for all interviews and three independent researchers for data analysis ensured quality control and minimal interpretive bias.

Despite the small number of interviewees, with nine interviews conducted, the high information power of the data allowed for the achievement of the aim of this study [58]. Due to a strict time schedule of conducting the study, no comparisons between the experiences of the women in different parts of Germany were aimed for, which would have required a bigger sample including,for example, more women living in isolated regions.

There is the possibility that the sample in our study included mostly women with more negative experiences of health-seeking in Germany, who might have been more motivated to talk about these experiences and therefore participate in our study. In addition, this study does not account for the experiences of women with limited access to health insurance as in the situation of newly arrived refugees. A potential limitation of our study might be that we reported the experiences of the women in the healthcare setting, but do not know what took place from the viewpoint of the healthcare providers.

Therefore, caution should be taken in generalizing the results to the experiences of all Syrian migrant women in Germany as bigger (quantitative) studies would be necessary to verify the results found in this explorative qualitative study.

## Conclusions

This exploratory study provides in-depth insights into the experiences and needs of Syrian immigrant women regarding managing their health and that of their families after their resettlement in the community in Germany. Providing tailored information on the German healthcare system, creating a health-literacy supportive environment, and improving cultural sensitivity in healthcare provision could help Syrian women better utilize medical care offered in Germany and better manage health situations. The highly evident mental distress experienced by the women in this study alerts to the need for targeted policies and services to address the mental health of Syrian immigrant women after their resettlement in the German society. Further research is needed to explore how the resilience chances for this group could be better incorporated into health and integration efforts in a culturally-sensitive and empowering way.

## Supplementary Information

**Additional file 1:** Interview guide.

## Data Availability

The data generated and/or analysed during the current study are not publicly available due to restrictions related to confidentiality i.e. they contain information that could compromise the privacy of research participants, but are available from the corresponding author SK on reasonable request.

## Abbreviations

IP: Interviewed Person
OECD: The Organisation for Economic Co-operation and Development
REFUGIUM: Rat mit Erfahrung: Flucht und Gesundheit – Information und Multiplikation (Advice with experience: Health and refugium_ information and multiplication)
MiMi: Mit Migranten für Migranten, interkulturellen Gesundheitsförderung Projekt (From immigrants to immigrants, intercultural health promotion project)
UPD: Unabhängige Patientenberatung Deutschland (Independent Patient Counselling Germany

## Footnotes

1 Refugee Health Awareness and Empowerment Program in Hamburg, Germany (48).

2 Mit Migranten für Migranten, interkulturellen Gesundheitsförderung Projekt: from immigrants to immigrants, intercultural health promotion programme for immigrants in Germany (49).

3 Independent Patient Counselling Germany (UPD gGmbH) (37).
