# Supplementary material for: Exploring how Syrian women manage their health after migration to Germany: results of a qualitative study
Source: BMC Womens Health. 2021 Feb 2;21:50. doi: 10.1186/s12905-021-01193-9 (PMC7852358; doi:10.1186/s12905-021-01193-9)
Supplement: Supplementary file 1 — Additional file 1: Interview guide. [file 12905_2021_1193_MOESM1_ESM.docx]

| Theme | Theoretical sub-themes | Question |
| --- | --- | --- |
| Health in Germany | - Perceived Health - Attitude towards ill-health - Language | 1. **How has your health (and that of you family) been since you have moved to Germany?** 2. **Can you think of a time when you felt unwell (or one of your children was ill) here in Germany, what did you do?** 3. **How does the language affect your ability to be healthy here in Germany?** |
| Health as an immigrant woman | - Social role - Difficulties - Facilitations - A process | 1. **How do did your life as a woman change after moving to Germany? How does this effect your health?** 2. **As an immigrant woman in Germany, what are the difficulties that you face here when it comes to situations of your health or that of your family?** 3. **If we compare your life here to that in Syria, are there any changes that made you more capable in handling your health and that of your family?** **Are there any changes that made you less capable?** 4. **How did this change over time (different points in the migration process: when you were living in the refugee camp, when you moved to your own housing, when you started working, when you had your child, etc.)** |
| Abilities and competences | - Accessing healthcare - Dealing with the healthcare system - Controlling health on a daily basis - A process | 1. **How do you feel about your ability to access health care when needed?** 2. **How do you feel about your ability to deal with papers and procedures related to healthcare here in Germany?** 3. **How do you feel about your ability to lead a healthy life here in Germany?** 4. **How did this change over time (see question 7).** |
| Resources | - Social support - Money - A process | 1. **If you need help or there is something you don’t know about your health (or that of your family) here in Germany, who would you turn to?** 2. **How does money (financial issue) affect your ability to be healthy?** 3. **How did this change over time?** |
| Knowledge and information | - knowledge of health rights - knowledge of women rights - knowledge about health | 1. **Do you feel that you understand your health rights and options in the Germany?** 2. **What would you do if you are not satisfied with the healthcare you receive here?** 3. **How do you feel about your knowledge of your legal rights as a woman here in Germany? Did you experience any changes to Syria in relation to gender equality?** 4. **Where do you get your information about health? How do you judge whether it’s correct or not?** |
| Influence | - Culture - Social influences | 1. **Are there any cultural or religious factor that affect the way you make health decisions?** 2. **What influence do others have on your decisions about your health?** |
| Participation | - Choice of participation in (health) and social activities | 1. **Do you take part of any organized social activities here in Germany?** 2. **As you might have noticed, here in Germany, the focus is on prevention and improving health through lifestyle modification, how do you experience this in your life here in Germany?** |
| Future health decisions | - Reflections on future health decisions | 1. **in what ways do you feel you can make better health decisions for you and for your family in the future?** 2. **What do you think is necessary for you to make better decisions about your health in the future?** |

**Additional file 1: Interview guide**
